# A Novel Biomarker of Compensatory Recruitment of Face Emotional Imagery Networks in Autism Spectrum Disorder

**DOI:** 10.3389/fnins.2018.00791

**Published:** 2018-11-01

**Authors:** Marco Simões, Raquel Monteiro, João Andrade, Susana Mouga, Felipe França, Guiomar Oliveira, Paulo Carvalho, Miguel Castelo-Branco

**Affiliations:** ^1^Coimbra Institute for Biomedical Imaging and Translational Research, Instituto de Ciências Nucleares Aplicadas à Saúde, University of Coimbra, Coimbra, Portugal; ^2^Faculty of Medicine, University of Coimbra, Coimbra, Portugal; ^3^Center for Informatics and Systems, University of Coimbra, Coimbra, Portugal; ^4^Neurodevelopmental and Autism Unit from Child Developmental Center, Hospital Pediátrico, Centro Hospitalar e Universitário de Coimbra, Coimbra, Portugal; ^5^PESC-COPPE, Universidade Federal do Rio de Janeiro, Rio de Janeiro, Brazil; ^6^University Clinic of Pediatrics, Faculty of Medicine of the University of Coimbra, Coimbra, Portugal; ^7^Centro de Investigação e Formação Clínica, Hospital Pediátrico, Centro Hospitalar e Universitário de Coimbra, Coimbra, Portugal

**Keywords:** emotional facial expression, mental imagery, EEG biomarker, machine learning, autism spectrum disorder, dynamic expressions

## Abstract

Imagery of facial expressions in Autism Spectrum Disorder (ASD) is likely impaired but has been very difficult to capture at a neurophysiological level. We developed an approach that allowed to directly link observation of emotional expressions and imagery in ASD, and to derive biomarkers that are able to classify abnormal imagery in ASD. To provide a handle between perception and action imagery cycles it is important to use visual stimuli exploring the dynamical nature of emotion representation. We conducted a case-control study providing a link between both visualization and mental imagery of dynamic facial expressions and investigated source responses to pure face-expression contrasts. We were able to replicate the same highly group discriminative neural signatures during action observation (dynamical face expressions) and imagery, in the precuneus. Larger activation in regions involved in imagery for the ASD group suggests that this effect is compensatory. We conducted a machine learning procedure to automatically identify these group differences, based on the EEG activity during mental imagery of facial expressions. We compared two classifiers and achieved an accuracy of 81% using 15 features (both linear and non-linear) of the signal from theta, high-beta and gamma bands extracted from right-parietal locations (matching the precuneus region), further confirming the findings regarding standard statistical analysis. This robust classification of signals resulting from imagery of dynamical expressions in ASD is surprising because it far and significantly exceeds the good classification already achieved with observation of neutral face expressions (74%). This novel neural correlate of emotional imagery in autism could potentially serve as a clinical interventional target for studies designed to improve facial expression recognition, or at least as an intervention biomarker.

## Introduction

Faces represent a critical source of visual information for social perception, conveying relevant information about identity and emotional states of others (Kanwisher and Yovel, 2006). Since the first months of life, children are capable of understanding and processing facial cues, like FEs (Field et al., 1982). The ability to interpret these social signs represents an essential skill in child development and, therefore, a basic condition for the development of the ability to engage in successful social interactions early in life (Bayless et al., 2011).

ASD is a neurodevelopmental disorder characterized by deficits in the social domain which represent hallmark early characteristics (Sperdin et al., 2018). Even for simple visualization of FE, the literature is somewhat inconsistent: while some studies show group differences both in behavioral performance and neural responses, other studies show no identifiable deficits at all (for a compreensive review, see Monteiro et al., 2017).

Importantly, no previous study has considered the role of MI in the FE processing domain, possibly because of the challenges in identifying imagery signatures that mimic neural responses during simple observation. The perceptual strength and spatial frequency of the FE stimuli seem to be relevant to yield ASD group differences during simple visual presentation (Vlamings et al., 2010; Luckhardt et al., 2017), but the large majority of visual perception studies use static frame stimuli, lacking the dynamic characteristics of naturalistic FE (Monteiro et al., 2017). Those dynamics have been shown to play a crucial role on the perception of the respective FE and its emotional valence (Krumhuber et al., 2013) possibly because they allow to generate perception and action imagery cycles.

Another limiting aspect is the notion that specific processing experimental contrasts are needed to isolate effects of interest. For example, the use of blank screen baselines, before the presentation of faces, generates a non-specific contrast of face with expression against a baseline without any stimulus. Therefore, those responses comprise both the processing of low-level core aspects of the face and the specific processing of the FE. In this EEG study we used dynamic FE morphing in a virtual avatar and used its neutral expression as baseline, to ensure a FE specific contrast. This way, the neutral FE is already present in the baseline. We believe this stringent contrast provides a response specific to the processing of the FE aspects, isolating it from the simple response to the face static itself. A systematic review of EEG studies regarding FE processing in ASD conducted by Monteiro et al. (2017) has already identified the need for experimental paradigms targeting the dynamic characteristics of FEs. All the studies identified by that review applied non-specific experimental contrasts, using blank screens as baseline of their experimental conditions. To the best of our knowledge, our study is the first one to combine a task-specific contrast for dynamic FE stimuli.

MI is defined as the simulation or re-creation of perceptual experience (Kosslyn et al., 2001; Pearson et al., 2013). Most of these mental representations are extracted from memory and allow one to mentally revisit the original stimuli or their combination (Pearson et al., 2015). Disturbed MI has been postulated to be present in several psychiatric disorders, from post-traumatic stress disorder (Lanius et al., 2002) to socio-emotional disorders like social phobia or depression (Hirsch et al., 2006). In the specific case of ASD, MI is likely to be impaired, since one of the key deficits included in the ASD diagnosis, in the form of absence or impairment of ‘pretend play’ (Baron-Cohen et al., 2001; American Psychiatric Association [APA], 2013), which requires preserved action-perception imagery cycles. This deficit is particularly interesting since it spans into the social, imitation and repetitive behavior dimensions (Crespi et al., 2016). Therefore, the study of the neural correlates of MI in ASD gains relevance since it might lead to the understanding of the neural correlates of its core neurodevelopmental limitation and further help into the development of successful therapies.

Here, by providing a critical link between visual observation and subsequent replay imagery, we bound MI to the FE of an avatar, in a task where the participant mentally replays the previously observed dynamic image of the avatar performing a happy or a sad FE. We believe this link between visual observation and MI of FE in others addresses both the deficits of FE processing, emotion identification and theory of mind, due to the lack of thinking from the perspective of the other present in ASD. Therefore, the concept of visually imagining others smiling recruits the faculties of expression processing and pretend play, and our experimental design allowed to study such imagery process in ASD, and to use two distinct classification approaches, based on linear and non-linear features describing brain signals, to differentiate between the disease state and normal cognition. Non-linear features consist of quantitative measures that represent in a relatively simple way complex dynamic characteristics of the EEG signals, which the traditional linear methods (amplitude and frequency, for example) are not able to capture. They have been adopted more and more frequently in EEG analysis in general and ASD biomarker research in particular (Bosl et al., 2011, 2017).

## Materials and Methods

### Participants

Seventeen male teenagers with the diagnosis of idiopathic ASD were recruited from the Unit of Neurodevelopment and Autism from the Pediatrics Unit from the University Hospital of Coimbra and from Portuguese ASD patient associations (Coimbra and Viseu). Since ASD is a disorder far more prevalent in male individuals, with a ratio of four males to every female, and there is accumulated evidence for sex differences in brain connectivity (Alaerts et al., 2016; Irimia et al., 2017; Fu et al., 2018), only male participants were included in the study. The diagnosis of ASD was performed based on the Autism Diagnostic Observation Schedule, the Autism Diagnostic Interview – Revisited and the Diagnostic and Statistical Manual of Mental Disorders – 5th edition criteria, confirmed by an expert multidisciplinary team. Seventeen healthy TD male controls were recruited from our local database of volunteers. Participants from both groups had their IQ assessed by the Wechsler Adult Intelligence Scale for participants older than 16 years old, and by the Wechsler Intelligence Scale for Children for younger participants. Groups were matched by chronological age (ASD mean age and standard error (SE): 16.4 ± 0.6 years; TD mean age and SE: 15.5 ± 0.6 years) and performance IQ (ASD mean score and SE: 99.8 ± 3.0; TD mean score and SE: 106.2 ± 4.2). Additional group characterization can be found in Table 1.

**Table 1:** Group characterization: mean and standard error of the mean (between brackets) of age, full scale IQ (FSIQ), verbal IQ (VIQ) and performance IQ (PIQ) (^∗^*p* > 0.05).

	ASD	TD	
N	17	17	
Age	16.4 (0.6)	15.5 (0.6)	^∗^
FSIQ	92.2 (3.1)	109.2 (4.5)	
VIQ	88.1 (4.2)	110.3 (4.2)	
PIQ	99.8 (3.0)	106.2 (4.2)	^∗^


Written informed consent was obtained from the parents of the participants or, when appropriate, the participants themselves. The study was approved by the ethics committee from Faculty of Medicine from the University of Coimbra and was conducted in accordance with the declaration of Helsinki.

### Experimental Tasks

The experiment is divided in two tasks: one of visual stimulation and one of MI requiring “mental replay” of previously observed FE, with the goal to identify similar neural signatures. The visual stimulation task and overall experiment were developed in *WorldViz Vizard 5 VR Toolkit* (development edition) using the *male002* virtual avatar from the *Complete Characters HD pack* and its FE poses. The total duration of the experiment is about 50 min, including 15 min for scalp cleaning and placement of the EEG cap, 30 min for the experimental tasks and 5 min to clean up at the end of the session.

#### Visual Stimulation Task

**FIGURE 1:**
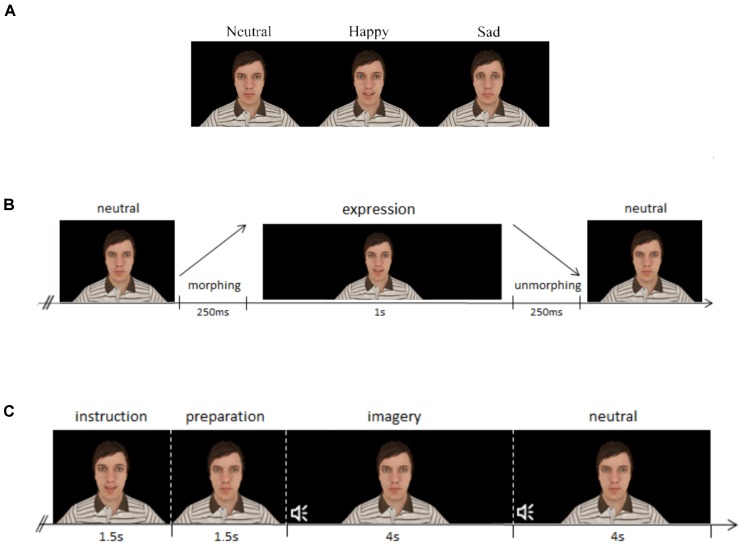
Description of the tasks, both regarding structure, and stimuli used. **(A)** Base stimuli used for each expression at their expression endpoint, comprising the neutral, happy, and sad facial expressions. **(B)** Structure of the visual stimulation paradigm: each expression lasted 1.5 s, divided by facial expression morphing (250 ms), static facial expression (1 s) and facial expression unmorphing (250 ms). **(C)** Structure of the mental imagery paradigm: the instruction is composed by the avatar performing the expression to be imagined, as presented in the visual stimulation task, and to facilitate mental replay. After that, an interval of 1.5 s is left for preparation, and an auditory stimulus (beep) cues the start of the mental imagery process, for 4 s, whereas another beep indicates the end of the mental imagery of the expression, and the start of the neutral period.

This task consists in observing a virtual avatar performing either sad or happy FEs (see Figure 1A), which represent two antagonistic expressions from the six core expressions (Ekman and Friesen, 1971). The FEs were verified in accordance with the action units defined in the Facial Action Coding System (FACS) (Ekman and Friesen, 1978). The happy expression comprises action units 6 (cheek raiser), 12 (lip corner puller) and 25 (lip part), while the sad FE uses action units 1 (inner brow raiser), 2 (outer brow raiser), 4 (brow depressor), 15 (lip corner depressor), and 17 (chin raiser).

Each trial is composed by a morphing period of 250 ms where the expression of the avatar gradually changes from neutral to the target expression, followed by a static period where the virtual avatar is displaying the target FE for 1000 ms and a final period where the avatar morphs back to the neutral expression, with the duration of 250 ms (see Figure 1B). Thus, each stimulus has a duration of 1.5 s and the inter-trial interval consisted in 1s plus a jitter of 500 ms. The neutral face of the avatar is always present during the baseline/inter-trial interval, which creates a stringent contrast with the FE since the stimuli does not come from no stimulus/blank screen, but from the neutral face, as naturally happens in real life.

This part of the experiment is composed by two blocks of 120 randomized trials (60 of each FE), for a total of 240 trials. The participants were asked to fixate the face of the avatar in the middle of the eyes and observe the expressions. A rest period was included between blocks to ensure focus and reduce fatigue throughout the experiment. A total of 120 trials per condition were recorded.

#### Mental Imagery Task

The second part of the experiment consists of a MI paradigm. In this task, the participant is asked to mentally imagine the avatar performing the same types of FEs used in the stimulation part (used to facilitate mental replay). The computer screen shows the neutral face of the avatar during the whole period, except for the instruction, when it performs the FE the participant is asked to imagine. Then, after a cue, the participant imagines the avatar performing the FE, in a period of 4 s, returning to no imagery after that period. The (c) section of Figure 1 details the structure of the trials. This task is composed by two blocks of 40 randomized trials (20 for each expression), achieving a total of 80 trials for the task.

### Experimental Setup and Data Recording

The experiment was conducted in a 22-inch LCD Monitor (frame rate of 60 Hz, 1680 × 1050 pixel resolution). The participants sat about 60 cm away from the screen (distance measured from the eyes to the center of the screen) and were asked to keep their eyes open and fixed on the face of the avatar. EEG data were recorded using a 64 channel actiCHamp system from Brain Products.

The scalp of the participants was first cleaned using abrasive gel and then the 64 channel actiCAP cap was placed on their head. Data were recorded from 64 Ag/AgCl active electrodes (Brain Products), placed across the head according to the international 10–10 standard system. The ground electrode was placed at AFz position and the reference electrode at the right ear. The impedance of the electrodes was kept under 10 kΩ during the recordings. The electrodes were connected directly to the Brain Products actiCHamp amplifier and sampled at 1000 Hz. EEG data were recorded using the Brain Products Recorder software. For each paradigm, the individuals were informed about the respective task. The total duration of the experimental procedure (preparation + 2 tasks) was around 50 min.

### EEG Preprocessing

We used MathWorks Matlab, 2017b and the EEGLAB toolbox v14.1.1 (Delorme and Makeig, 2004) for EEG signal preprocessing and analysis. EEG data were filtered with a finite impulse response bandpass filter of frequencies 1 and 100 Hz and notch filtered with an infinite impulse response filter between 47.5 and 52.5 Hz, as implemented in the EEGLAB toolbox. Bad channels were removed and data were re-referenced for the average reference. Epochs were created locked to the stimulus onsets (please refer to the task-specific analysis for details about the epoch lengths). Bad epochs were removed based on the EEGLAB semi-automatic procedures for extreme values and improbable signal segments. Independent Components Analysis (ICA) was then run on the data using EEGLAB implementation of *infomax* algorithm (Bell and Sejnowski, 1995). Components were used in order to extract noisy components, such as blinks, muscular activity or electrical interference. Components presenting such artifacts were removed and the weights were projected back to the data (Makeig et al., 2004). Bad channels previously removed were then interpolated. Further analysis of EEG data was conducted over these preprocessed signals.

### Experimental Design and Statistical Analysis

The analysis focused on identifying group differences for both visualization and MI of the FEs. We specify the different analyses performed for each task separately.

#### Visual Stimulation Task Analysis

The visual stimulation epochs comprise 1 s, starting 100 ms prior to the stimuli onset (baseline) and go to 900 ms after the start of the expression morphing (during the first 250 ms of the epoch, the face of the avatar is continuously morphing the FE). ERPs were computed by subtracting each epoch by the mean of its baseline (from 100 ms pre-stimulus to 0) and then averaging all epochs corresponding to the same stimulus condition.

Source analysis were conducted using the sLORETA toolbox (Pascual-Marqui, 2002). The procedure included exporting from EEGLAB the preprocessed single-trial epochs, importing them into sLORETA software, averaging them (per subject and expression) and converting to the source space. Each participant electrode locations were co-registered with the realistic anatomical MR model using landmarks and standard electrode positions. The source space representation consists of a current source density (CSD) map computed with the sLORETA algorithm, a standardized discrete three-dimensional (3D) distributed linear weighted minimum norm inverse solution that takes several neurophysiologic and anatomical constraints into account and has been shown to yield depth-compensated zero localization error inverse solutions (Pascual-Marqui, 1999; Pascual-Marqui et al., 2002). sLORETA employs the current density estimate given by the minimum norm solution, and localization inference is based on standardized values of the current density estimates (Pascual-Marqui, 2002) and has been shown to outperform its competitor algorithms in terms of localization error and ghost sources (Grech et al., 2008).

For each expression and each group, we identified the peaks of the first and second ERP component for each electrode, and extracted the latencies for both peaks across the scalp. We performed the source localization of the mean activity of around those two ERP components (±125 ms, see Supplementary Figure S1).

We conducted a voxel-by-voxel between-group comparison of the mean current source density distribution in those time windows around the ERP peaks, using the sLORETA software implementation of SnPM, employing a log-*F*-ratio statistic for independent groups (for a similar procedure see, for example, Velikova et al., 2011). The SnPM method corrects for multiple comparisons without requiring Gaussian assumptions (Nichols and Holmes, 2001).

#### Mental Imagery Task Analysis

For the MI task, we also performed ERP analysis locked to the sound trigger. For the longer imagery blocks, we performed a spectral source analysis at more distant time windows and investigated the statistical classification of putative neural biomarkers.

##### Mental imagery ERP source analysis

For the imagery epochs, we investigated the ERP sources originated by the happy and sad imagery triggers. The participant receives the instruction beforehand of which expression to imagine. We segmented the trials from 100 ms prior to the cue beep and up to 900 ms after it, and subtracted them by the mean of their baseline (-100 ms to 0).

Similarly to the visual stimulation ERPs, for the source analysis we looked for the mean global field power in the window of 0–250 ms. The pipeline was analogous to the VEP, as well as the statistical framework.

##### Mental imagery spectral source analysis

For the MI periods, we investigated frequency bands of the signal during the time window of 500–3500 ms, avoiding the contribution of the beep ERP and covering the main period of MI, because MI processes are best captured using time-frequency analysis (Horki et al., 2014). The frequency bands of interest were θ, α, β, and δ, as defined in the sLORETA toolbox. This analysis of frequency bands of induced activity comprised the following steps: we export the single trials from EEGLAB and imported them to the sLORETA toolbox. Then we compute the cross-spectrum of each trial and average them per subject and condition. The average cross-spectrum is used to compute the source current density maps used in the second-level analysis.

For both ERP and frequency analysis we conducted voxel-by-voxel between-group comparisons of the current density distribution for each expression, in a way analogous to the VEP procedure.

##### Mental imagery biomarkers to classify groups

To explore the MI processes through the EEG data, we defined several features from the time, frequency, and non-linear domain. We then performed a ranking analysis and selected the best features to train a classifier to discriminate participants between groups. Features were extracted for each channel and trial by trial and averaged across all imagery trials and electrode clusters.

###### Feature extraction

We follow the procedure of Simoes et al. (2015) for extracting features representative of different EEG characteristics.

###### Time/frequency domain

For the time and frequency domain, we selected the signal envelope (env), Teager energy operator (teag) and instantaneous power (pow) as features. A detailed description of these features is present in Supplementary Table S1.

###### Non-linear domain

To extract signal complexity measures, the EEG signal was transformed to its phase-space. The phase-space is a reconstruction of the chaotic dynamics of the system and, as was proven by Takens (1981), it keeps some of the relevant properties of the state space representation of the system, such as the topographic properties, Lyaponov exponents and the Kolmogorov-Sinai Entropy. Every possible state of the system can be represented by a point in the multidimensional phase space and time evolution of the system creates a trajectory in the phase space (Kliková and Raidl, 2011). We used the time delay method to reconstruct the phase-space of the signal. Given a time series of a scalar variable it is possible to construct a vector _X(t_i_), i=1, …,N_ in phase-space in time *t_i_* as follows:

$$X(t_{i})=[x(t_{i}),x(t_{i}+\tau ),...,x\left(t_{i}+\left(m-1\right)\tau \right)],i=1,...,N-(m-1)\tau$$

where τ is time delay, *m* is the dimension of reconstructed space and _M=N-(m-1)τ_ is the number of points (states) in the phase space.

We reconstructed a 2 and 3-dimensional phase-space associated to the EEG data, and the time delay was considered to be the mean of the first local minimum from the signal’s autocorrelation (hereafter defined as lag).

From the non-linear domain we extracted the spatial filling index (SFI), largest Lyapunov exponent (Lyap), correlation dimension (CorrDim), approximate entropy (ApEn) and sample entropy (SpEn) as features. We provide a detailed description of these features in the Supplementary Table S2.

The features were extracted from 3 time windows in each trial: baseline [-500 ms to 0 ms pre instruction], emotion imagery [500–3500 ms after imagery trigger] and neutral [500–3500 ms after neutral trigger]. For the emotion and neutral time windows, we used the absolute value for the non-linear features and the normalized values (subtracted by the same feature extracted from the baseline) for the time/frequency domain.

###### Frequency bands

All features were extracted from signals filtered at different frequency bands. Band-pass Infinite Impulse Response (IIR) filters were used as implemented in EEGLAB toolbox, for the frequency bands: θ [4–8] Hz, α [8–12] Hz, low β [12–21] Hz, high β [21–30] Hz and γ [30–40] Hz.

###### Feature selection

In order to reduce the dimensionality of the feature set, we averaged the features extracted from each electrode in spatial clusters, as defined in Supplementary Figure S2. The clusters were defined by electrode spatial proximity in a way that covers the full scalp, keeps symmetry and lobule divisions (frontal, parietal – subdivided in central and posterior region, occipital and temporal). We then used the *a priori* information provided by the source localization and selected only the clusters closer to the right precuneus region, namely C1, C2, C4, and C5.

We ended up with 8 different features × 5 frequency bands × 4 clusters, for a total of 160 different features. We then computed the statistical discriminative value of each feature between groups with two sample *t*-tests, using only the samples from the training set, and the features were ordered by absolute T value, from the most important to the least.

###### Classification

We trained a SVM with a linear kernel, for being one of the most used classifiers applied to EEG signals (Lotte et al., 2007) and also a WNN. The WNNs are underused in the literature but present characteristics that generalize well for noisy domains, like the EEG (Simões et al., 2018a). We implemented a variation of the WiSARD combined with a bleaching technique (França et al., 2014) which has been shown to perform at the same level as the SVM in distinct fields and presents fast learning curves, achieving good results even with small datasets of data (Cardoso et al., 2016).

We trained the classifiers to discriminate the group of the participant, based on the feature vector extracted from his EEG data. We divided the participants into train and test sets: 80% of the cases were randomly chosen for training and the remaining 20% for testing. We repeated the procedure more than 30 times, to avoid overfitting, following the guidelines provided by Varoquaux et al. (2017) regarding the use of machine learning on brain imaging data. Feature selection was performed every time using only training-set data.

To explore the relation between accuracy and the number of features used, the procedure was conducted starting with 5 features and adding 5 more features up to the total of features.

We repeated the full classification procedure using the EEG signal from the neutral part of the MI task, in order to check if the results were specifically improved during over emotion expression imagery.

## Results

### Visual Stimulation Task

This section presents the results of the analysis performed on the ERP responses to the visual stimulation task (observation of happy and sad FEs), which was used to identify neural signatures relevant to validate the imagery task.

#### ERP Source Analysis Results

**FIGURE 2:**
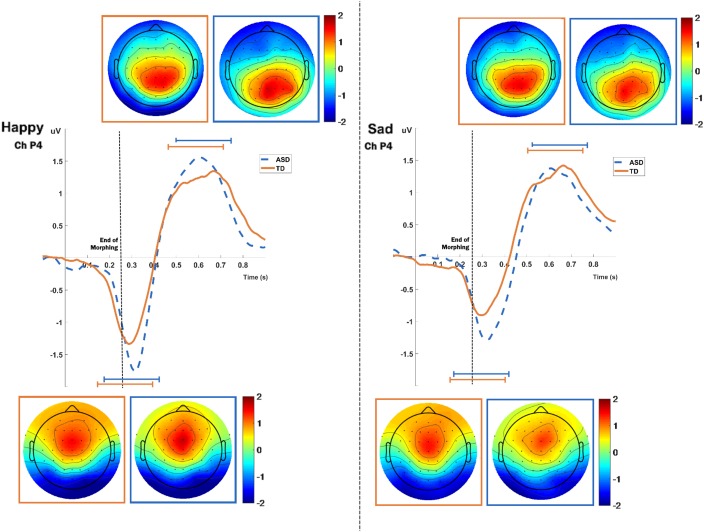
ERPs for both groups and expressions, extracted from the P4 electrode. Topographic maps for each component are present near the ERP plots. Orange marks represent the TD group and blue marks the ASD. Topographic maps show the scalp distribution of the ERP amplitudes extracted from 250 ms windows, centered at the peaks of the components of each expression (refer to Supplementary Table S3 for detailed peak latencies).

The ERPs obtained from the visual stimulation task present two clear independent components, the first one peaking around 300 ms and the second around 600 ms (Figure 2). Since the morphing occurs during the first 250 ms, we expect a delay on the first component, as reported by Graewe et al. (2012). The topography of the first component matches the well-known topography of the N170 component, with a negativity around the right and left parietal-occipital regions, but it appears delayed in time, as expected by the morphing animation. The second component has a strong parietal positivity, slightly right lateralized, especially for the ASD group.

For the source analysis of the visual stimulation task ERPs we defined time-windows of 250 ms around the two component peaks of activity in the ERPs. We show the results for the first and the second ERP component, separately. The mean peak latencies used for each expression and each group is detailed in Supplementary Table S3.

**FIGURE 3:**
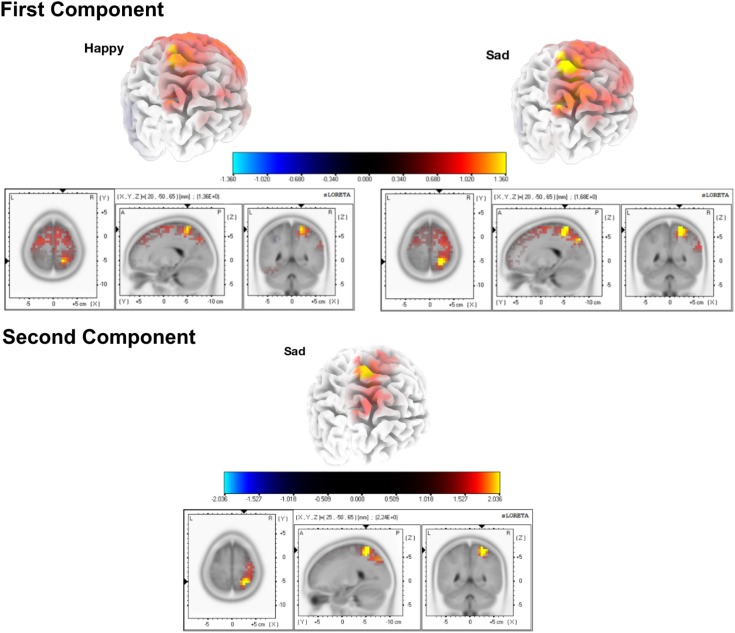
Source group differences for the first and second ERP components, for happy and sad expressions. We found higher activation for the ASD group in the right precuneus using a two tailed alpha level of 5%, corrected with the SnPM method. Regarding the second component, this result was statistically significant specifically for the sad expression.

The mean current source density of activity in the intervals around the component peaks showed group differences for both expressions in the first component, using voxel-by-voxel independent tests between groups, corrected for multiple comparisons at the 5% level using the SnPM method (two-tailed). Both expressions show the group differences right-lateralized and located at the superior parietal region, in the precuneus area (Figure 3). As for the second component, only the sad expression presented statistically significant differences, exactly in the same superior parietal region, which showed also enhanced recruitment for the ASD group, in the right hemisphere.

### Mental Imagery Task

This section presents the results for the MI task. We analyzed the ERP for the initial imagery period and the longer MI blocks through source analysis of the power spectrum and the analysis of several characteristics of the signal using machine learning techniques.

#### Mental Imagery ERP Source Analysis Results

**FIGURE 4:**
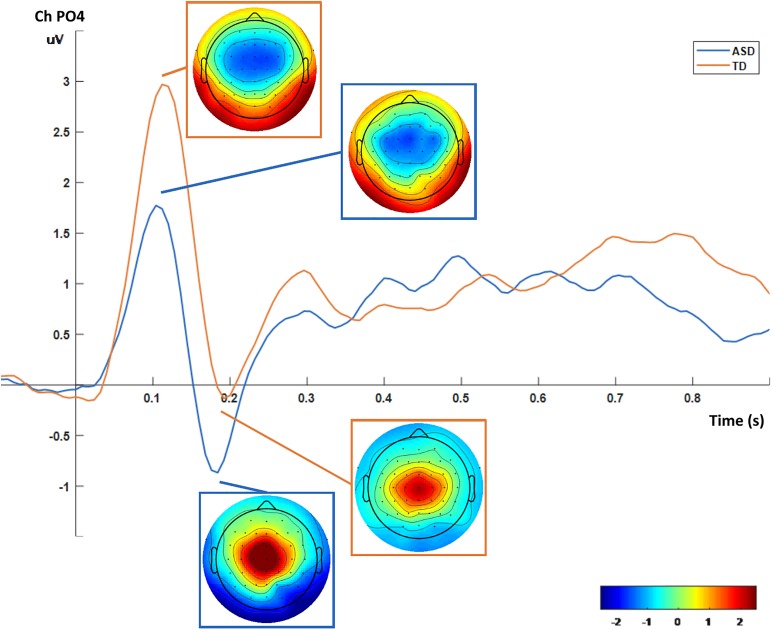
ERP and topographic plots for the mental imagery task (PO4 channel). An initial ERP is visible peaking positively at 100 ms and negatively at 200 ms, with the tonic spectral characteristics overtaking the remaining time period (from 0.5 s onward).

**FIGURE 5:**
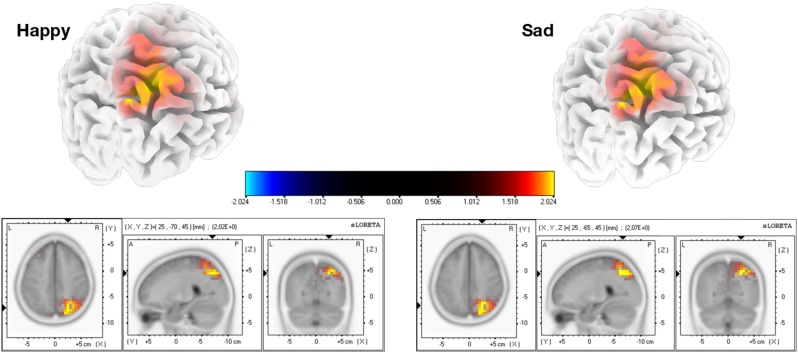
Group differences for the source analysis of the ERPs of mental imagery. Statistical differences (two-tailed *p* < 0.01, SnPM corrected) were found in the region of precuneus, with higher activation for the ASD group.

After the sound trigger, an initial ERP can be found corresponding to processing the beep and starting the imagery procedure (Figure 4). We defined a time window to target at the source level, between 0 and 250ms, in order to investigate specific responses at the source level. The mean current source density in that interval presented group differences for both expressions with *p* < 0.01, using voxel-by-voxel independent tests between groups, corrected for multiple comparisons using the SnPM method. Importantly, the same region identified group differences for both expressions. This was also the same region that was identified during visual stimulation. Accordingly, the ASD group presented higher activation in the superior parietal region (precuneus area – Figure 5).

#### Mental Imagery Spectral Source Analysis Results

**FIGURE 6:**
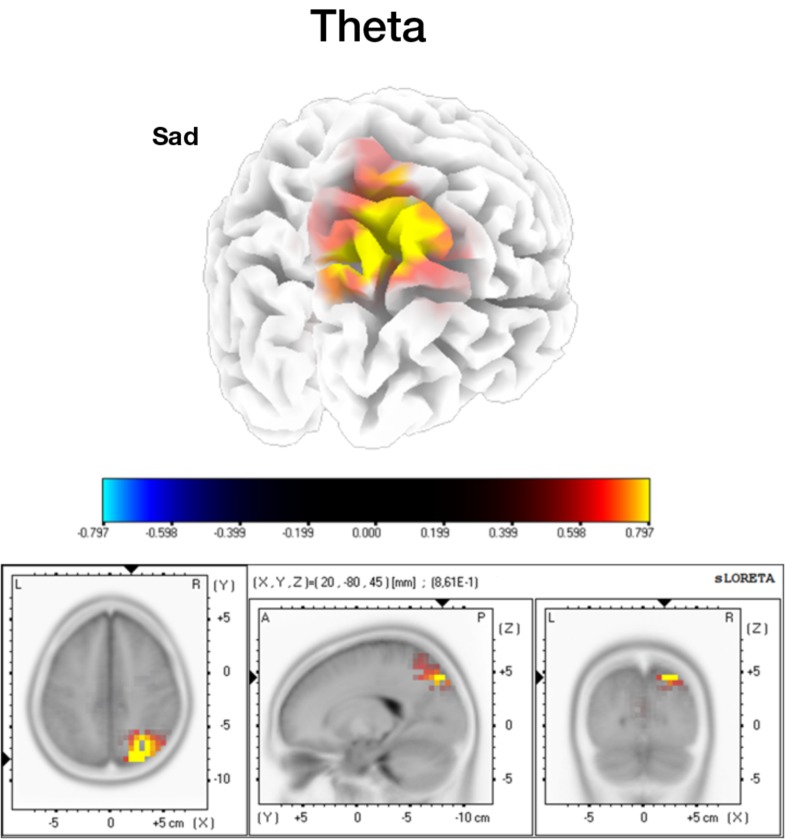
Source analysis for the mental imagery segments, in the theta band. Higher activation for the ASD group in the precuneus area (two tailed *p* < 0.05, SnPM corrected).

For the longer periods of imagery (500–3500 ms), we conducted a source analysis of the defined frequency bands of the signal. A statistical significant result was found for in the imagery of sad expressions, for the theta band (Figure 6). The ASD group shows again higher recruitment of the very same right precuneus area at this frequency.

#### Statistical Classification of Mental Imagery Periods – Evidence for a Potential Biomarker in ASD

**FIGURE 7:**
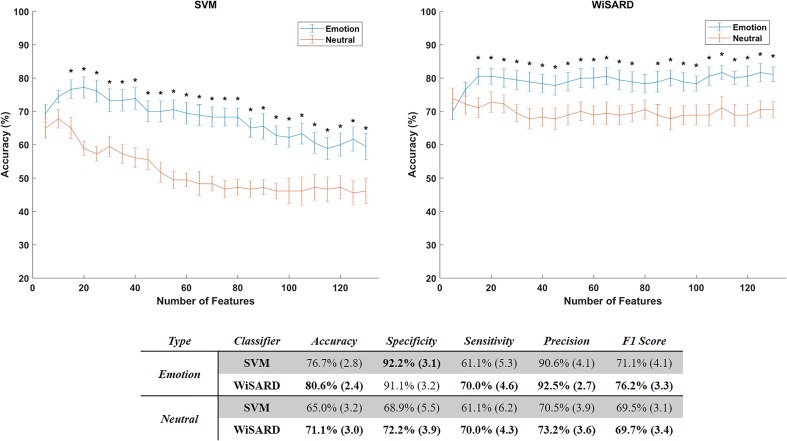
Accuracy of the classifiers SVM (left) and WiSARD (right) as function of the considered number of features. Mean accuracies are represented with the lines and the error bars show the standard error of the mean. Classification results with the mental imagery part of the EEG signals are represented in blue and the neutral signals in orange. Statistically different accuracies between Emotion and Neutral are marked by ^∗^ (one-sample *t*-tests with alpha level of 5% and false discovery rate correction for multiple comparisons). At the bottom we present the performance metrics for both classifiers using the top 25 features. Each cell presents the mean values followed by the standard error of the mean of the respective metric.

We then tested whether the identified neural signatures of imagery of FEs could be identified in a data driven manner using statistical classifiers. The linear SVM and the WiSARD classifier were able to achieve high test set accuracies (∼77% and ∼81% of accuracy, respectively), with the WiSARD yielding the best accuracy of 81% with just 15 features (Figure 7). Test set classification accuracy of the neutral face expression segments of the signal were far worse, with ∼68% for the SVM and ∼74% for the WiSARD, suggesting that important group differences are captured by the features are emotion expression-dependent (for statistical details see Figure 7). We present also a detailed exploration of the performance metrics using the top 15 features. We computed accuracy, specificity, sensitivity/recall, precision and the F1 score for both classifiers using the MI segments and the neutral segments.

We checked the correlation value between the extracted features and the IQ measurements (full-scale, verbal and performance IQ), and no feature was significantly correlated with any of the covariates.

**FIGURE 8:**
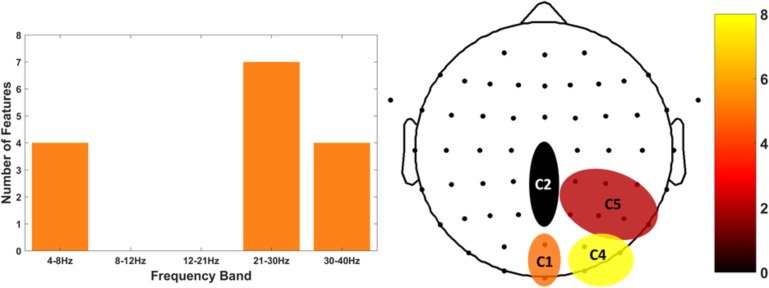
Top 15 features distribution by frequency band (left) and clusters (right). The histogram on the left depicts the exploitability of theta and high-beta/gamma frequency features. The histogram of the right shows the scalp distribution of features within the right parietal-occipital region, showing a preference for the posterior clusters of the region.

We then focused on the top 15 features that generated the 81% of accuracy. We investigated the most selected frequency bands and clusters of these top features. Figure 8 shows the top 15 feature distribution by clusters and frequency bands, showing the specific contribution of theta, high beta and gamma bands for group discrimination. Detailed feature information (Table 2) clarifies that the most discriminative features originate from the time-frequency domain, at the high-beta/gamma bands, and that the non-linear features are mainly from the theta-band.

**Table 2:** List of the top 15 features used in the classifiers, showing their frequency band, cluster, and statistical value.

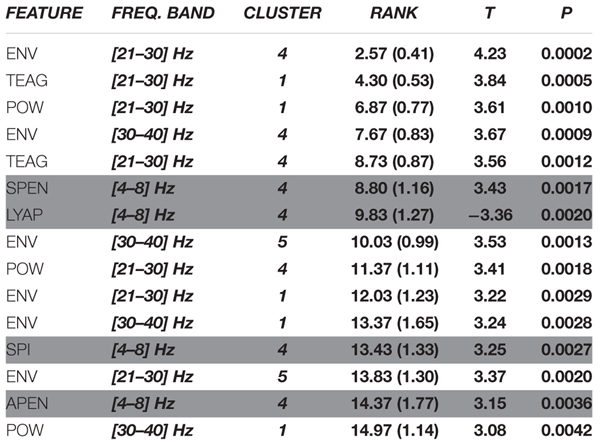

*Non-linear features are presented with gray background and time/frequency features with white background. Rank values correspond to the mean order of the feature across training sets, with the respective standard error of the mean. T and P values for each feature are presented, resulting from an independent *t*-test between the groups. All the 15 features are statistically significant (corrected for multiple comparisons using the false discovery rate algorithm).*

## Discussion

Here we addressed for the first time FE imagery in ASD and identified a common neural correlate of observation and MI of dynamic FEs in this condition, in the precuneus. Robust statistical classification of brain activity patterns using linear and non-linear features could also be achieved, and the identified biomarker of abnormal imagery in ASD can potentially be used as an outcome measure to evaluate clinical interventions addressing cognitive and behavioral improvement in this condition.

We focused on MI of FEs in ASD as a major research target in this study. This is a very important cognitive process in the context of this disease, because mental rehearsal is very important for action perception cycles, in particular in the context emotional face recognition. MI is the process of creating a mental representation and corresponding sensory experience of an episode or stimulus without a direct external source (Pearson et al., 2015). In the case of FEs, it also involves MI of motor patterns (FEs) which requires the involvement of the mirror neuron system. There are indeed several types of MI, namely visual, auditory and motor (for a review, see Kosslyn et al., 2001). Some studies showed the effect of MI on boosting performance in detection tasks (Tartaglia et al., 2009) and on decision making bias (Pearson et al., 2009). In our study, participants were asked to perform visual MI of an avatar performing a FE (mentally replaying previously observed patterns). This task combines MI, perspective taking and theory of mind, since the participant is asked to recreate an expression of another.

A critical aspect that renders the study of imagery difficult in ASD is that it is important to ascertain that imagery really reflects the expected visual content. We could achieve this by showing that similar neural signatures (source localization) can be found by both observation and imagery of FEs. The ERP elicited by the imagery cue did indeed reveal that source differences were very similar as compared to the ERP of the FE stimuli, with the precuneus showing higher activation for the ASD group. The right precuneus belongs to task-active networks (Yang et al., 2015) that are also active during imagery [for a review of the relation with the precuneus with visuo-spatial imagery and visuomotor transformations, please refer to (Cavanna and Trimble, 2006)].

One of the common aspects of visualization and MI of the others FEs is the need to incorporate the perspective of the other. Because we use a stringent contrast in the visual stimulation task, we expected the core processing of the face to have less weight than the perspective taking aspects task. The precuneus is one of the core regions present in the perspective taking network, as showed by Healey and Grossman (2018). The authors reviewed the literature and found the precuneus as a key region in both cognitive and affective perspective taking networks (Abu-Akel and Shamay-Tsoory, 2011). Those fMRI studies validate the source we identified in our study.

The link between the precuneus and its role in FEs processing has already been demonstrated by some studies (Saarimäki et al., 2016; An et al., 2018), but our study is the first one, to the best of our knowledge, to identify the over-recruitment of this region in the ASD population in a social cognition task. Since visual perspective taking and theory of mind skills are impaired in ASD (Hamilton et al., 2009; David et al., 2010), we believe that ASD participants needed higher recruitment of the right precuneus as a compensatory mechanism for the MI of the other’s FE.

Frequency band decomposition of the MI signals showed that theta and high-beta/gamma bands explained the main group differences. The source analysis of the theta band further revealed again a higher activation of the right precuneus for the ASD group (specifically for the sad FE). It was already known that FEs elicited higher theta responses than neutral expressions in healthy participants (for a review, please refer to Güntekin and Başar, 2014). Although theta band activity patterning has been linked to the medial frontal cortex and its role in cognitive control (Cavanagh and Frank, 2014) its source in our study seems to be different. In agreement with our own source, Wang et al. (2016) demonstrated a relationship between the theta band and activity patterns in the posterior cingulate cortex/precuneus, in a simultaneous EEG-fMRI study. Furthermore, the study from Knyazev et al. (2009) identified the same right parietal source from theta responses to FEs. Therefore, we believe the parietal theta band relation with the precuneus to be a core neural correlate of emotional MI processing. Despite using different types of signals (phasic or tonic in relation to the type of mental process) to perform the source localization (ERP and time-frequency decomposition), due to the characteristics of the tasks, it is very interesting to observe the same region involved in both visualization and MI processes.

The precuneus is recruited in several types of imagery, including motor imagery, mental navigation, memory-related imagery, episodic source memory retrieval and emotional state attribution (Cavanna and Trimble, 2006). Specifically regarding attributing emotions to others, several studies identified the role of the precuneus in Theory of Mind scenarios (Vogeley et al., 2001; Takahashi et al., 2015). Moreover, a connectivity analysis study of resting state fMRI data showed decreased connectivity of the precuneus region with the middle temporal gyrus and the ventromedial frontal cortex in the ASD population, in both hemispheres (Cheng et al., 2015). All these observations pinpoint the precuneus as playing a pivotal role in FE MI. Furthermore, the group difference in the right hemisphere, which is also known to dominate in attention and imagery, suggests that the ASD group processes the FEs of the other in a more effortful, attention-based mechanism than the TD group. This view has been suggested by Harms et al. (2010). Our study is the first one, to our knowledge, to show that the same neural pattern that is observed during FE recognition is replicated for MI of the FEs, in ASD.

Based on the observed group differences, we investigated whether we could extract features that would function as biomarkers (not necessarily as diagnostic, but as intervention targets) of ASD, based on the MI process. The need for diagnostic, prognostic and intervention biomarkers in ASD is well recognized. While ASD biomarkers range from genetics to clinical (for a review, please refer to Ruggeri et al., 2014), the inter-subject variability observed in this disorder justifies the use of machine learning techniques combining multiple features to generate potential biomarkers (Huys et al., 2016). Therefore, we developed two classifiers – a SVM and a WNN to classify each subject (represented by a feature vector extracted from his EEG data) into ASD or TD group. Our purpose is to show that the features used by the classifiers provide exploitable group differences, that can also be used to characterize neural mechanisms underlying ASD (in this case, FE processing) and therefore be used to monitor, for example, rehabilitation efficacy (outcome measure) or aid at subgroup stratification in the ASD population (Castelhano et al., 2018), albeit not for early detection.

We verified that the WNN method achieved around 81% of accuracy using 15 features. When compared to the same classifiers trained with features extracted from EEG of the neutral periods, the accuracy was significantly lower (around 73%).

We then performed a further analysis of the top 15 features selected for classification. The most representative frequency band, when using non-linear features, was the theta band, while the most discriminative features were from the time/frequency domain and high-beta/gamma frequency bands. Those bands and their relation with the precuneus have been explored in the literature by Fomina et al. (2016), which attempted to train the self-regulation of gamma and theta bands in the precuneus in amyotrophic lateral sclerosis patients. This is consistent with our results, showing that the precuneus activity at the theta and high-beta/gamma bands represent important MI information that can be used for clinical purposes, for instance in BCI based neurofeedback.

The overall use of dynamic FE morphing enabled a more realistic and ecologic approach, because the stimuli featured more realistically the daily life characteristics of social interactions than the commonly used static stimuli. Moreover, we used a specific face expression contrast (emotional expressions vs. neutral expression). As stated by Krumhuber et al. (2013), the dynamic characteristics of FEs are possibly also understudied which is a limitation for the validity of neurocognitive approaches.

Our approach to morph the expression into a virtual avatar makes a potential bridge between dynamic FEs and rehabilitation possibilities using, for instance, virtual reality. Understanding how the FEs are processed in virtual environments opens the door for intervention solutions, where the environment is completely controlled (Miller and Bugnariu, 2016; Simões et al., 2018b). This is important because the neural markers identified in this study could potentially be used as intervention target measures.

A common characteristic of most studies in the literature using EEG and observation of FEs is the use of a blank-screen as baseline for the visual stimulus (Monteiro et al., 2017), thus eliciting ERPs that mix the processing of the FE with face and other non-specific visual features. We argue that the use of a more specific contrast (expressionless/neutral face as baseline) elicits an ERP specific to the dynamic expression characteristics of the face, not the face itself. Moreover, Monteiro et al. (2017) demonstrate disparate findings in the literature when evaluating EEG responses to FEs in ASD. Several studies found expression effects accompanied by group effects. Using a very specific contrast, we were able to identify, even for FE observation, group differences in the right precuneus, with the ASD group showing higher activation in this region. The functional role of precuneus in attentional deployment and imagery is well recognized (Cavanna and Trimble, 2006), with some studies also suggesting a relation to perspective taking (Vogeley et al., 2001; Kircher et al., 2002; Schurz et al., 2015), face familiarity (specifically for the left precuneus) (Lee et al., 2013) and emotional state recognition and attribution (Ochsner et al., 2004; Spies et al., 2017). Our right precuneus group effect for both happy and sad expressions is consistent with several studies using fMRI that reported the same effect for ASD in the right precuneus (see the meta-analysis of Aoki et al., 2015, which found hyperactivation of bilaterate thalamus, caudade and right precuneus for the ASD group). Especially in tasks requiring taking the others perspective, the recruitment of the precuneus is key in both cognitive and affective perspective taking networks (Healey and Grossman, 2018). We hypothesize that the ASD group performs a higher recruitment of the precuneus region to compensate for emotional processing and perspective taking behavioral deficits.

Our study focused only on male subjects to avoid an effect of gender in the analysis. There is evidence for sex differences in brain connectivity in ASD which might influence the EEG analysis we conducted (Alaerts et al., 2016; Irimia et al., 2017; Fu et al., 2018). The replicability of these results in female ASD cohorts lacks further validation. Moreover, in spite of the limitations of our sample size, it paves the way for future replication studies in larger groups.

In conclusion, we found for the first time, a neural correlate of emotion expression imagery in ASD, which was validated as a replication of the neural signatures evoked by visual observation of specific FEs. We developed an innovative approach to study FE processing in ASD, combining visualization of dynamic FEs (with a very selective contrast, isolating pure FEs from the mere presence of a face) and MI of FEs in others. Our results emphasize the important role of the precuneus in the ASD facial processing circuit and suggest that its increased recruitment may serve as a compensatory strategy to overcome the natural deficits in their emotional processing. Furthermore, we extracted a set of features and trained a classifier that was able to discriminate between groups with high accuracy. The features were then observed to match topographically and spectrally the group effects, and can therefore be potentially used as intervention targets.

## Data Availability

The raw data supporting the conclusions of this manuscript will be made available by the authors, without undue reservation, to any qualified researcher.

## Ethics Statement

This study was carried out in accordance with the recommendations and Guidelines of Comissão de Ética da Universidade de Coimbra, with written informed consent from all subjects. All subjects gave written informed consent in accordance with the Declaration of Helsinki. The protocol was approved by the Comissão de Ética da Universidade de Coimbra.

## Author Contributions

MS, RM, JA, SM, GO, PC, and MC-B conceived and designed the study. MS, RM, and JA performed the experiments with the participants. SM performed the screening and psychological evaluation. MS, RM, and JA analyzed the data. MS, RM, JA, PC, FF, and MC-B discussed the results and iterative approaches. MS, RM, and MC-B wrote the paper. All authors read, contributed and approved the final manuscript.

## Conflict of Interest Statement

The authors declare that the research was conducted in the absence of any commercial or financial relationships that could be construed as a potential conflict of interest.

## Abbreviations

ASD: autism spectrum disorder
ERP: event-related potential
FE: facial expression
fMRI: functional magnetic resonance imaging
IQ: intelligence quotient
MI: mental imagery
sLORETA: standardized low resolution brain electromagnetic tomography
SnPM: statistical non-parametric mapping
SVM: support vector machine
TD: typically developed
VEP: visual evoked potential
WiSARD: wilkes, stonham and aleksander recognition device
WNN: weightless neural network
