# Supplementary material for: A Novel Biomarker of Compensatory Recruitment of Face Emotional Imagery Networks in Autism Spectrum Disorder
Source: Front Neurosci. 2018 Nov 1;12:791. doi: 10.3389/fnins.2018.00791 (PMC6221955; doi:10.3389/fnins.2018.00791)
Supplement: Supplementary file 1 [file Table_1.DOCX]

**Supplementary Table 1 - Time / Frequency domain features and their description**

| Code | Feature Name | Description |
| --- | --- | --- |
| Env | Signal envelope | Envelope of the signal (smooth curve outlining the signal extremes), which corresponds to the magnitude of the analytic signal. The analytic signal is composed by the original waveform and its Hilbert transformation. Hilbert transformation of the signal corresponds to the original waveform with a 90º phase shift. Mathematically, the analytic signal z(t) is defined by  $z\left( t \right)=x\left( t \right)+\mathrm{ix}^{'}\left( t \right)$ (1)  in which i represents $\sqrt{-1}$, x’(t) corresponds to the Hilbert transformation of the original signal x(t) (Sadjadi and Hansen, 2015) |
| Teag | Teager energy operator | An energy estimation operator which uses the sum of the instantaneous energy of the signal divided by the signal length. The instantaneous energy of a signal x at the sample n can be determined using the equation  $\Psi\left[ x_{n} \right]= x_{n}^{2}-x_{n-1}x_{n+1}$ (2)  where $\Psi\left[ . \right]$ denotes the Teager energy operator (Solnik *et al.*, 2010). This operator is sensitive to both amplitude and frequency. |
| Pow | Instantaneous power | The instantaneous power is achieved by squaring its values  $P[x(t)] = x^{2}(t)$ (3)  where P$\left[ . \right]$ denotes the instant power of the signal x at the instant t. |
