# Supplementary material for: A Novel Biomarker of Compensatory Recruitment of Face Emotional Imagery Networks in Autism Spectrum Disorder
Source: Front Neurosci. 2018 Nov 1;12:791. doi: 10.3389/fnins.2018.00791 (PMC6221955; doi:10.3389/fnins.2018.00791)
Supplement: Supplementary file 2 [file Table_2.DOCX]

**Supplementary Table 2 - Non-linear domain features and their description**

| Code | Feature Name | Description |
| --- | --- | --- |
| SFI | Spatial Filling Index | The states are normalized to the interval [-1, 1]. The phase space area is then divided into small square areas of size $R\times R$, and the number of grids in the normalized phase-space is $n=[2/{R]}^{2}$. A new matrix can be obtained with its elements equal to the number of phase space points falling in each grid (Faust *et al.*, 2004). Spatial Filling Index corresponds to the probability of a phase space point falling in a grid. |
| Lyap | Largest Lyapunov Exponent | Characterizes the rate of separation of infinitesimally close trajectories of the signal in phase space, providing a measure of the degree of the system’s instability (Cencini *et al.*, 2010). Mathematically, two trajectories in the phase space with initial separation of $\delta Z_{0}$, diverge at a rate given by  $\left\vert\delta Z(t) \right\vert\approx e^{\lambda t}\left\vert\delta Z_{0} \right\vert$ (5)  where $\lambda$ is the local Lyapunov exponent (local exponential rate of expansion) (Cencini *et al.*, 2010). The rate of separation can be different for different orientations of initial separation vector, leading to a spectrum of Lyapunov exponents – equal in number to the dimensionality of the phase space. The maximum Lyapunov exponent corresponds to the mean exponential rate of divergence, characterizing the trajectory’s instability (positive values are associated with a chaotic system). |
| CorrDim | Correlation Dimension | Chaotic dynamic systems exhibit strange attractors, which tend to be self-similar. The Correlation dimension is a measure of the space fractal dimensionality of the attractor defined in the phase space. Correlation sum is defined as sum the fraction of pairs of points of the phase space whose distance is smaller than r, being r the Lag (defined previously). If this number of points is sufficiently large, the ratio between the logarithm of the correlation sum and logarithm of the time delay is a good estimate of the Correlation Dimension (Cencini *et al.*, 2010). |
| ApEn | Approximate Entropy | Quantifies the amount of the regularity and unpredictability of fluctuations of the signal. A time series with many repetitive patterns has a small value of ApEn, reflecting its predictability; the opposite happens for less predictable signals (Yentes *et al.*, 2013). |
| SpEn | Sample Entropy | This feature is a modification of ApEn used for assessing the complexity of a physiological time series data. ApEn depends on the length of the time series and lacks relative consistency. SpEn, similarly to ApEn, quantifies the regularity of the signal but does not have the aforementioned disadvantages (Yentes *et al.*, 2013). |
