# Supplementary material for: A Novel Biomarker of Compensatory Recruitment of Face Emotional Imagery Networks in Autism Spectrum Disorder
Source: Front Neurosci. 2018 Nov 1;12:791. doi: 10.3389/fnins.2018.00791 (PMC6221955; doi:10.3389/fnins.2018.00791)
Supplement: Supplementary file 3 [file Table_3.DOCX]

***Supplementary Table 3 - Latencies of the mean ERP components and respective standard error (in brackets), for each group and facial expression.***

| Group | Expression | First Component | Second Component |
| --- | --- | --- | --- |
| ASD | Happy | 295 (±9) ms | 579 (±16) ms |
|  | Sad | 299 (±15) ms | 585 (±15) ms |
| TD | Happy | 265 (±10) ms | 492 (±14) ms |
|  | Sad | 285 (±15) ms | 576 (±19) ms |
