# Supplementary material for: A Novel Biomarker of Compensatory Recruitment of Face Emotional Imagery Networks in Autism Spectrum Disorder
Source: Front Neurosci. 2018 Nov 1;12:791. doi: 10.3389/fnins.2018.00791 (PMC6221955; doi:10.3389/fnins.2018.00791)
Supplement: Supplementary file 4 [file Data_Sheet_1.PDF]

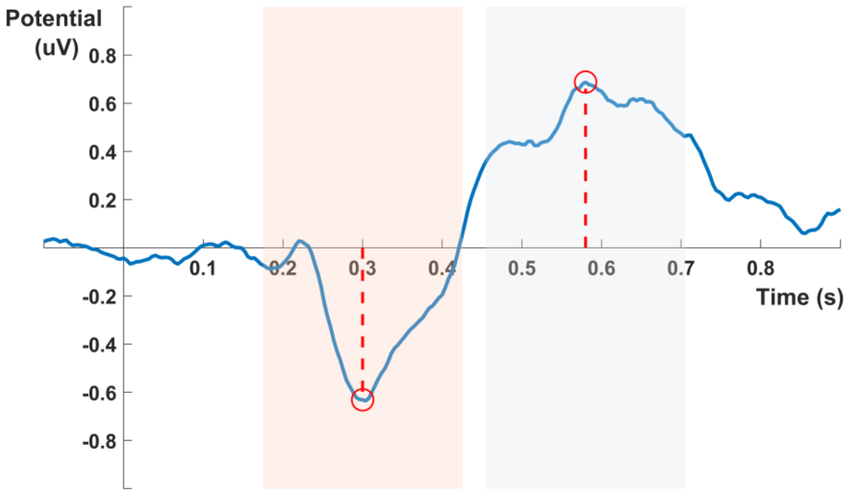

Supplementary Figure 1 - ERP component windows of interest, defined by the minimum and maximum value of the ERP of each electrode.
