# Supplementary material for: A Novel Biomarker of Compensatory Recruitment of Face Emotional Imagery Networks in Autism Spectrum Disorder
Source: Front Neurosci. 2018 Nov 1;12:791. doi: 10.3389/fnins.2018.00791 (PMC6221955; doi:10.3389/fnins.2018.00791)
Supplement: Supplementary file 5 [file Data_Sheet_2.PDF]

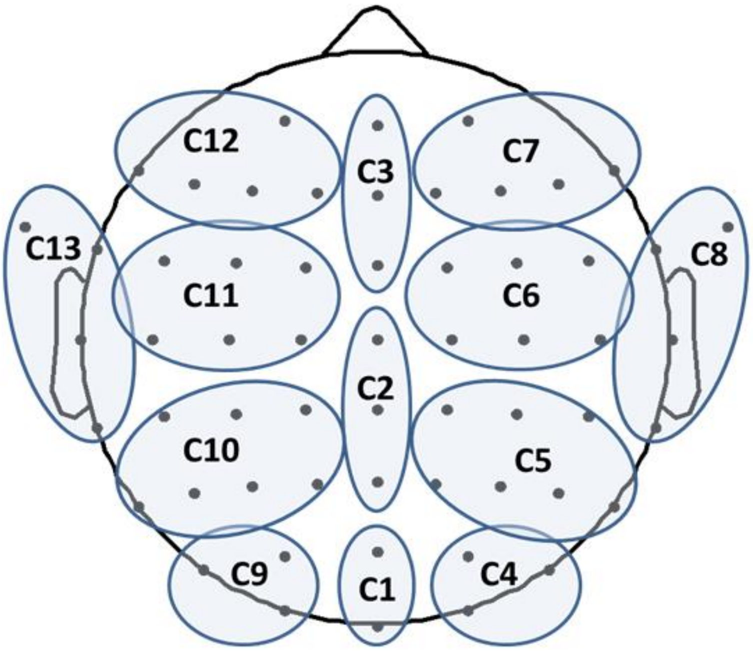

Cluster	Electrodes
C1	POz Oz
C2	Cz CPz Pz
C3	AFz Fz FCz
C4	PO4 PO8 O2
C5	P2 P4 P6 P8 CP2 CP4 CP6
C6	C2 C4 C6 FC2 FC4 FC6
C7	F2 F4 F6 F8 AF4
C8	FT8 FT10 T8 TP8
C9	O1 PO3 PO7
C10	P1 P3 P5 P7 CP1 CP3 CP5
C11	C1 C3 C5 FC1 FC3 FC5
C12	F1 F3 F5 F7 AF3
C13	TP7 T7 FT7 FT9

Supplementary Figure 2 - Clusters defined for the analysis. A full scalp distribution of the clusters was created in order to keep left and right occipital, parietal, central, frontal and temporal areas, and three central clusters for frontal, parietal and occipital lobes. Channel locations are represented with the 10-10 system standard codes.
